# Developing a low back pain guideline implementation programme in collaboration with physiotherapists and chiropractors using the Behaviour Change Wheel: a theory-driven design study

**DOI:** 10.1186/s43058-024-00568-x

**Published:** 2024-04-03

**Authors:** Maja Husted Hubeishy, Camilla Blach Rossen, Petra Dannapfel, Kristin Thomas, Tue Secher Jensen, Thomas Maribo, Nanna Rolving

**Affiliations:** 1 Interdisciplinary Research Unit, Elective Surgery Centre, Silkeborg Regional Hospital, Hospital in Central Denmark Region, Falkevej 1-3, 8600 Silkeborg, Denmark; 2 Department of Public Health, Aarhus University, Aarhus, Denmark; 3 Department of Physiotherapy and Occupational Therapy, Aarhus University Hospital, Aarhus, Denmark; 4 Department of Health, Medicine and Caring Sciences, Linköping University, Linköping, Sweden; 5 Diagnostic Centre – Imaging Section, Silkeborg Regional Hospital, Silkeborg, Denmark; 6 Department of Sports Science and Clinical Biomechanics, University of Southern Denmark, Odense, Denmark; 7 DEFACTUM, Central Region Denmark, Aarhus, Denmark

**Keywords:** Development, Implementation programme, Involvement, Behaviour Change Wheel, COM-B, Low back pain, Guidelines, Physiotherapists, Chiropractors

## Abstract

**Background:**

Low back pain is still the leading cause of disability and societal burden, with 619 million prevalent cases worldwide in 2020. Most countries produce clinical guidelines to support healthcare professionals in evidence-based care regarding low back pain. However, several studies have identified relatively poor uptake of guidelines. Tailored strategies to facilitate the implementation of guidelines have been argued to increase uptake. This study aimed to develop a contextually tailored implementation programme to enhance evidence-based low back pain care among Danish physiotherapists and chiropractors in primary care.

**Methods:**

A theory-driven implementation programme development study was conducted using the Behaviour Change Wheel, with high healthcare professional involvement. Data collection included four workshops with seven physiotherapists and six chiropractors from primary care clinics. The development process consisted of [1] establishing a theoretical frame, [2] involving participants, [3] understanding the behaviour, [4] designing the implementation programme, and [5] final implementation programme.

**Results:**

The target behaviours selected (guideline recommendations) for the implementation programme were (i) screening of psychosocial risk factors and (ii) offering patient education. The barriers and facilitators for the selected behaviours were described and linked to intervention functions and behavioural techniques. Finally, the implementation programme comprised five strategies: webinars, e-learning videos, communication exercises, peer learning, and group dialogue meetings. In addition, the programme consisted of implementation support: champions, a physical material folder, a weekly email reminder, a specially designed website and a visit from an implementation consultant. An essential element of the overall programme was that it was designed as a step-by-step implementation process consisting of 16 h of education and training distributed over 16 weeks.

**Conclusions:**

A programme for implementing low back pain guideline recommendations was developed based on behaviour change theory and four co-design workshops involving healthcare professionals to overcome the contextually identified barriers. A theory-driven approach involving healthcare professionals was useful in identifying relevant target behaviours and tailoring the programme to consider contextual barriers and facilitators for implementation. The effectiveness of the final implementation programme will be evaluated in the project’s next phase.

**Trial registration:**

**Central Denmark Region, Registered** November 11, 2021, act no. 1-16-02-93-19.

**Supplementary Information:**

The online version contains supplementary material available at 10.1186/s43058-024-00568-x.

Contributions to the literature • This study describes a theory-driven development process of an implementation programme to enhance evidence-based low back pain care among physiotherapists and chiropractors.

• The study contributes to *how* healthcare professionals can be involved in a development process using a co-design approach.

• The study presents a tailored step-by-step implementation programme for screening psychosocial risk factors and offering patient education in low back pain care.

• The study illuminates the black box of implementation mechanisms by providing a structured overview of how specific techniques can impact barriers and facilitators to behaviour change.

## Background

Low back pain (LBP) continues to be the leading cause of disability, with 619 million prevalent cases worldwide in 2020 [1, 2]. LBP is associated with a significant burden to patients, reporting that LBP has adverse effects on their social lives, emotional state, and ability to work [3]. Simultaneously, LBP is the condition for which the most significant number of people may benefit from evidence-based rehabilitation [4], a claim supported by a significant body of literature showing that the provision of evidence-based rehabilitation for patients with LBP leads to a decrease in healthcare utilisation compared to non-evidence-based care [5, 6]. To support healthcare professionals (HCPs) in providing evidence-based care, health authorities in most countries continuously produce and update clinical practice guidelines [7]. Although considerable effort and resources have been spent developing and updating these guidelines [8], several studies have identified relatively poor uptake of the guidelines by HCPs [9–15]. Thus, an opportunity exists to potentially optimise healthcare utilisation by effectively implementing LBP evidence-based practice [16, 17].

There is consensus that evidence-based care for patients with LBP should include screening of psychosocial risk factors, advice to stay or return to physical activity and work, exercise therapy, patient education including the provision of reassuring information regarding the benign nature and prognosis of LBP [18–21]. Also, psychosocial risk factors are broadly recognised to influence LBP occurrence and progression negatively, underlining the importance of implementing a biopsychosocial approach in clinical practice [3, 22, 23]. Nonetheless, screening patients’ psychosocial risk factors and patient education are challenges in clinical practice [24–27], thus hampering the implementation of the recommended biopsychosocial approach to LBP care. The main implementation barrier to applying the biopsychosocial approach, identified in the research literature, is a biomechanical professional identity, where the HCPs do not feel it is their role to provide a biopsychosocial approach and also feel a lack of knowledge, skills and confidence in using this approach [28–30]. Likewise, studies have found that the HCP approach is influenced by the public and patients’ perceptions and expectations of treatment based on a biomedical approach [28–30]. Another essential barrier is the longer treatment time a biopsychosocial approach requires.

Several studies have demonstrated that positive change in HCPs’ beliefs, attitudes, skills, awareness, and adherence to guidelines *can* be achieved through various implementation strategies [31–33]. However, to successfully implement and sustain evidence-based care, multi-pronged implementation strategies tailored to address multi-level, context-specific barriers and facilitators are required [34–38]. The chance of success can further be increased by involving the HCPs in developing the implementation programme for whom the behaviour change is intended [39–42]. In addition, implementation scientists agree that the development of implementation strategies should be guided by theory to promote implementation success [17, 34, 43]. For this purpose, theoretical models like the Behaviour Change Wheel (BCW) and the Capability, Opportunity, Motivation - Behaviour framework (COM-B) have been developed to guide interventions aiming for behaviour change [44, 45]. However, in most countries, the publication of guidelines lacks accompanying implementation strategies to ensure HCPs adopt the recommended practice change [46]. This insufficient attention to effective implementation strategies practice may explain the low uptake of these guidelines.

In Denmark, where the present study occurred, LBP guidelines are typically published on relevant websites, and professional organisations occasionally offer introductory meetings. However, no active implementation efforts are offered to support the HCPs in learning *how* to translate the guidelines into practice. Therefore, this study aimed to develop a contextually tailored LBP guideline implementation programme, informed by theory and in collaboration with physiotherapists and chiropractors, with the purpose of enhancing evidence-based LBP management in primary care.

## Methods

### Design

A theory-driven implementation programme development study using the Behaviour Change Wheel including COM-B [44] and participant involvement. Data collection included workshops with physiotherapists and chiropractors. The reporting of the study followed the GUIDED guideline (see Additional file 1) [47].

### Study and target population

Seven physiotherapists and six chiropractors from primary care clinics in the Central Denmark Region were recruited for participation (hereafter referred to as participants). To be eligible, participants had to work in a primary care clinic, manage at least one patient with LBP per week, and be able to participate in all workshops (dates were given in the invitation). To get multiple perspectives, a diversity in demographic variables like gender, geographic location (rural/urban), years of clinical experience, the size of the clinic, and level of adherence to guidelines (assessed in a survey conducted prior to this study) was aimed for in the sampling (see Table 1). In the selection, a search was made on www.sundhed.dk, a Danish website with an overview of all primary care clinics. Subsequently, each clinic’s website was reviewed, and a strategy was created to ensure diversity. Eligible participants were initially contacted via email and, if there was no response, by telephone calls after 7 days. An honorarium of €400 was provided in connection with participation in the study. The target group for the implementation programme was physiotherapists and chiropractors (hereafter referred to as HCPs) working in primary care clinics treating patients with LBP in all stages (acute, subacute, chronic).

**Table 1:** Characteristics of the participants

Variables	Chiropractors (*n*=6)	Physiotherapists (*n*=7)
Sex		
Male, *n* (%)	4 (67)	5 (71)
Years of clinical experience, mean (range)	17.5 (5–29)	21.4 (5–41)
Geographical location*		
Rural, *n* (%)	3 (50)	4 (57)
Urban, *n* (%)	3 (50)	3 (43)
Size of clinic**		
Small, *n* (%)	3 (50)	0 (0)
Medium, *n* (%)	3 (50)	6 (86)
Large, *n* (%)	0(0)	1 (14)

*Rural area defined as a population of less than 10,000, Urban area as a population of 10,000 or more

**A small clinic defined as a clinic comprising 1–4 healthcare professionals (HCPs), medium clinics comprised 5–9 HCPs, large clinics comprised 10 or more HCPs

### Setting and data collection

The design process consisted of four workshops, including thorough preparation of the workshops and data processing by the researchers between and after the workshops. The first two workshops took place at two of the participants’ clinics to mimic the context in which the implementation programme was to be used. Due to COVID-19, the third workshop was moved to the first author’s workplace at a Regional Hospital in Denmark, and the last workshop was held online. The first three workshops lasted 3.5 h, and the fourth workshop 2 h. The first author facilitated the workshops. All group discussions were audio-recorded, and some of the discussions were video recorded. Observation notes were taken during all the workshops by co-author CBR. All material produced by participants during workshops was also collected, e.g. post-its and posters, and considered as data in the development process.

### The development process

**Fig. 1:**
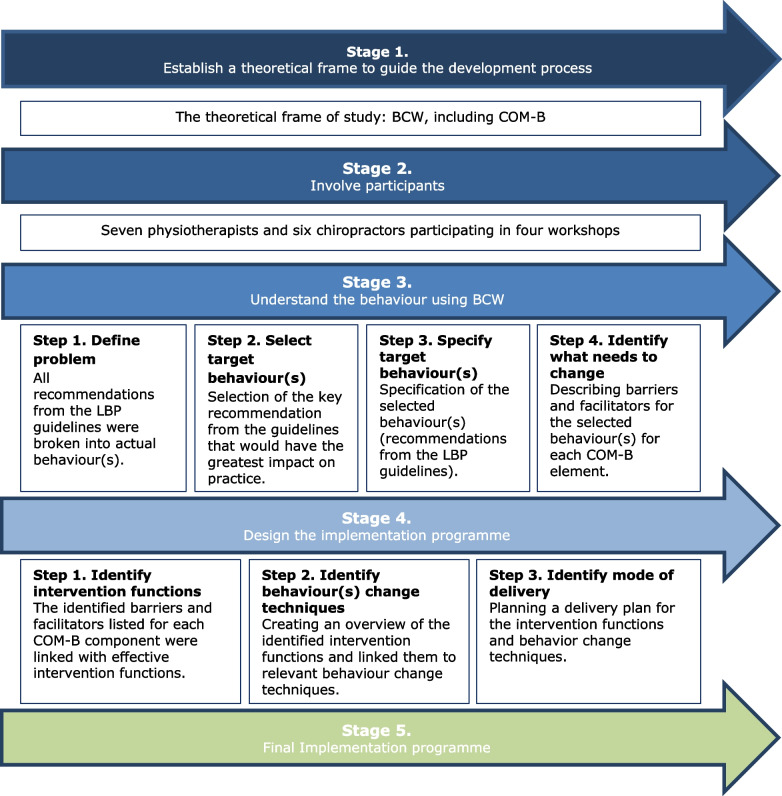
Development process of the implementation programme Legend: BCW: Behaviour Change Wheel, COM-B: Capability, Opportunity, Motivation – Behaviour, LBP: Low Back Pain

The development process consisted of five stages, in agreement with the recommended methodology for designing strategies to change behaviour [34, 43, 48]: 1) Establish a theoretical frame; 2) Involve participants; 3) Understand the behaviour; 4) Design the implementation programme; 5) Final implementation programme. Stages 3 and 4 each consisted of four and three steps, respectively. Figure 1 outlines the process of developing the implementation programme. The results section will present stages 3, 4 and 5.

#### Stage 1: Establish a theoretical frame to guide the development process

**Fig. 2:**
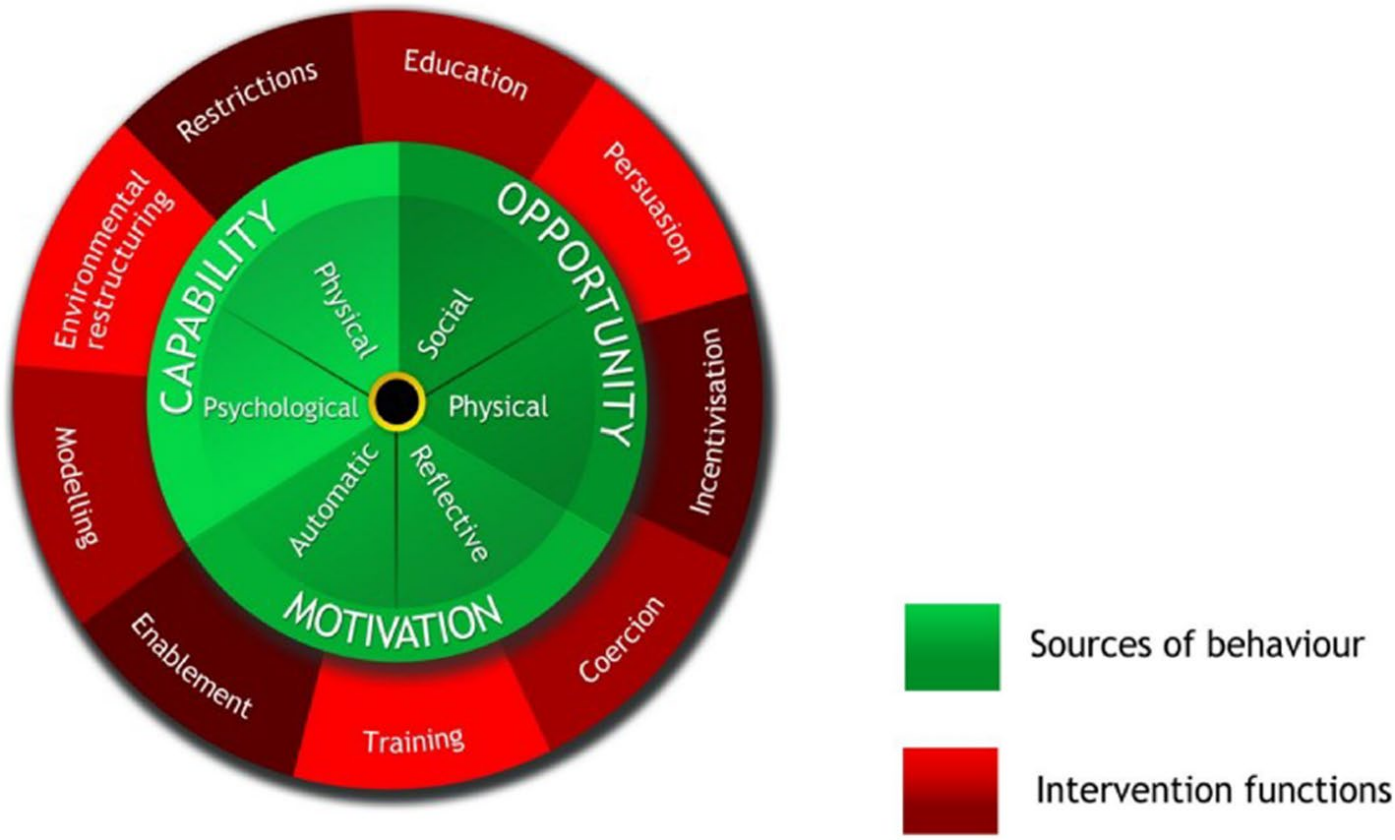
The Behaviour Change Wheel, including the COM-B components [44]

This study used the BCW, including the COM-B components. The BCW is a synthesis of 19 frameworks of behaviour change [44, 45], with the COM-B model being the starting point used by the BCW for understanding a given behaviour in the context in which it occurs (see Fig. 2) [44, 45]. COM-B describes three essential components for a behaviour to occur (green circle), namely capability, opportunity and motivation. Capability is the physical and psychological strength, such as the knowledge to perform a given behaviour. Opportunity is the conducive social and physical environment, e.g. sufficient time or facilities. Motivation is the reflective and automatic beliefs or reflex response to do the behaviour. BCW provides a systematic and theoretically guided method for identifying nine types of intervention functions (red circle) that would effectively change a given behaviour: Education, Persuasion, Incentivisation, Coercion, Training, Restrictions, Environmental Restructuring, Modelling and Enablement [45]. The BCW includes guidance on which intervention function is practical in a given COM-B domain. For example, if a barrier is identified as a lack of physical capability, linking training as an intervention function will likely be effective for a behaviour change.

#### Stage 2: Involve participants

The development of the implementation programme was co-designed using a participatory research approach [49, 50]. During four co-design workshops, participants were involved in the design stage, giving inputs to the content and delivery of the programme. At the first workshop, expectations were mutually matched, and roles and processes were clarified and balanced. The crucial role of the participants was to contribute to designing an applicable and appropriate implementation programme. The role of the participants was at different levels of involvement [51]. In parts of the design, they were active listeners; in other processes, they were advisors, e.g. advising on relevant barriers in the context, and in other processes, they were involved as decision makers, e.g. deciding which recommendations from the guidelines the programme should target. To ensure all participants were involved, they filled in written worksheets individually and were then divided into small groups, where everyone had the opportunity and time to have their say. The groups were divided by profession to clarify whether designing two strategies adapted to each profession was important. Every group work was followed up by group discussions, where the first author, MHH, focused on ensuring inclusivity and active participation from all participants, for example, by inviting more reserved participants to reflect on a question or present the worksheets in question.

#### Stage 3: Understand the behaviour using BCW and COM-B

According to the BCW, a crucial stage before designing the implementation programme is to understand the target behaviour. Stage 3 in the present study, therefore, consisted of four steps: 1) Define the problem to be addressed; 2) Select the target behaviour; 3) Specify the target behaviour; 4) Identify what needs to change.

##### Step 1: Define the problem to be addressed in behavioural terms (workshop 1)

The overall purpose of the implementation programme to be developed was to enhance the HCPs’ adoption of the LBP clinical practice guidelines [52, 53]. However, as “adherence to LBP clinical practice guidelines” is a broad term consisting of a series of behaviours, this step focused on breaking down all recommendations from LBP guidelines (see Table 2) into a number of more specific behaviours by the last author, NR, and co-author, TSJ, which were then presented at workshop 1.

**Table 2:** Overall recommendations from the Danish LBP clinical practice guidelines

**Assessment** • Screening of psychosocial risk factors • Do not offer routine imaging	
**Treatment** • Supervised training in addition to usual treatment • Manual joint mobilisation in addition to usual treatment • Do not offer routine acupuncture • Do not offer routine pain medication (paracetamol, NSAIDs, opioids)	
**Information and guidance** • Advise to stay physically active • Advise to stay or return to work as soon as possible • Offer patient education including reassuring information about the benign nature and prognosis of LBP	

##### Step 2: Select target behaviour(s) (workshop 1)

Acknowledging that it would not be realistic to implement all guideline recommendations described in Table 2, each participant prioritised which of the target behaviours (i.e. guideline recommendations) should be the focus of the implementation programme by filling out a worksheet consisting of the following questions:

1. Are the consequences of not applying the recommendation serious?
2. Are there many of your colleagues who do not use the recommendation?
3. Is the recommendation possible to apply in practice?
4. Is it important to implement the recommendation in practice?

All four questions had to be completed for all the recommendations in the guidelines on a scale of 1–5 (1: no, 2: probably not, 3: do not know, 4: probably yes, 5: yes). Each recommendation thus received a total score for all four questions (see Additional file 2). After completing the worksheet, the participants were divided into professional groups (physiotherapists and chiropractors), where they each had to present their total score and present and discuss their arguments for their selected target behaviours. Subsequently, the two groups were brought together to present their task results, and the two behavioural targets that had been given the highest priority were selected for the next step of the process in Workshop 2.

##### Step 3: Specify target behaviour in as much detail as possible (workshop 2)

At workshop 2, the participants were introduced to the 'video test', a method for specifying a target behaviour. The 'video test' requires the participants to describe the target behaviour so concretely that if someone were videotaped performing the behaviour, everyone would instantly know that he/she was performing it. The participants were then divided into small groups and asked to complete a second worksheet of four questions for each target behaviour: 1) What should the behaviour include? 2) How should the behaviour be practised? 3) When and how often should the behaviour be performed? 4) To whom should the behaviour be performed? (see Additional file 3). After the group meetings, the participants were gathered to share their experiences and thoughts.

##### Step 4: Identify what needs to change using COM-B (workshop 2)

After having specified the behaviour in step 3, the participants were divided into professional groups, where they completed a third worksheet with the following two questions, representing barriers and facilitators, for each chosen target behaviour: 1) What challenges does the new behaviour pose? 2) How may the new behaviour be supported? The two questions were to be completed for each component of COM-B (i.e. capability, opportunity and motivation). For example, for the target behaviour “screening of patients’ psychosocial risk factors”, the participants must write down barriers regarding psychological capability: Do the HCPs have the “psychological capability” such as knowledge and skills to screen the patients’ psychosocial factors? (see Additional file 4).

#### Stage 4: Design the implementation programme using BCW and COM-B

In alignment with the BCW, the target behaviour specified in stage 3 must be linked with intervention functions that serve as strategies for implementing the behaviour. In the present study, stage 4 consisted of three steps: 1) Identify intervention functions; 2) Identify behavioural change techniques; 3) Identify mode of delivery.

##### Step 1: Identify intervention functions (workshop 3)

In workshop 3, the participants were again divided into smaller groups divided by professions and asked to collectively complete a fourth worksheet, linking the identified barriers and facilitators with intervention functions. To support the linking process, the research team provided the participants with an overview of what intervention functions were applicable and effective for each barrier and facilitator in a given COM-B component. All participants then shared their thoughts on identified strategies in a structured plenum discussion led by the first author, MHH.

##### Step 2: Identify behaviour change techniques (between workshops 3 and 4)

Between workshops 3 and 4, MHH and NR created an overview of the identified intervention functions based on the fourth worksheet completed by the participants. Subsequently, they linked them to relevant behaviour change techniques. The behaviour change techniques described the components of the intervention functions at a more granular level.

##### Step 3: Identify mode of delivery (workshop 4)

At workshop 4, a delivery plan for the identified intervention functions and behaviour change techniques was discussed with the participants, including the following elements: Who should deliver? How should it be delivered? How often and over what period? Furthermore, it was discussed whether single elements should be pilot-tested before testing the implementation programme.

## Results

In the following, output from participant workshops from Stage 3 Understand the behaviour; Stage 4 Design the implementation programme; and Stage 5 Final implementation programme are presented.

### Stage 3: Understand the behaviour

#### The target behaviour selected

The recommendation regarding the screening of psychosocial risk factors received the highest score from both professions, with patient education scoring second highest (see Table 3). Both chiropractors and physiotherapists responded that the screening of psychosocial factors, particularly, was underutilised in practice, with scores of 3.6 and 4.4, respectively. Both groups recognised the importance of implementing the two recommendations and considered them necessary and feasible to implement in practice. However, the two professions only partially agreed on the ranking of the other recommendations. Following discussions, the two recommendations with the highest scores (recommendations mentioned above) were identified as the most critical behaviour targets for the implementation programme. Table 3 presents an overview of the various guideline recommendations and the scores assigned by the participants.

**Table 3:** Results from the first worksheet showing the selected recommendations

The recommendations in the guidelines	Are the consequences of not applying the recommendation serious?		Are there many who do not use the recommendation?		Is the recommendation possible to transfer into practice?		Is it important to implement the recommendation in practice?		Total score	
	CPs (*n*=6)	PTs (*n*=7)	CPs (*n*=6)	PTs (*n*=7)	CPs (*n*=6)	PTs (*n*=7)	CPs (*n*=6)	PTs (*n*=7)	CPs (*n*=6)	PTs (*n*=7)
Screening of psychosocial risk factors	4	4.6	3.6	4.4	4.6	4.4	4.4	4.9	16.6	18.3
Patient education	4.4	4.6	2.6	3.6	4.4	4.7	4.2	4.6	15.6	17.5
Supervised training	3.6	4.1	3.0	2.3	4.4	4.9	4.2	4.9	15.2	16.2
Advise to stay or return to work	3.8	4.6	2.8	3.0	3.8	4.1	4.6	4.7	15.0	16.4
Do not offer routine imaging	3.8	4.0	3.0	3.4	4.4	4.1	3.6	4.4	14.8	15.9
Manual joint mobilisation	3.8	3.0	2.4	2.4	4.2	4.3	4.2	3.9	14.6	13.6
Advise to stay physical active	4.0	4.7	1.2	2.1	4.2	5.0	4.4	5.0	13.8	16.8
Do not offer routine pain medication	3.2	4.1	3.4	4.1	3.2	4.0	3.0	4.5	12.8	16.7
Do not offer routine acupuncture	2.6	2.9	3.0	2.7	2.6	4.0	2.0	4.0	10.2	13.6

*CPs* chiropractors, *PTs* physiotherapists. Each number is an average score for each professional

The scores indicate 1: no, 2: probably not, 3: do not know, 4: probably yes, 5: yes. The total score is a summation of the 4 scores for each question

#### Specification of the target behaviour

**Fig. 3:**
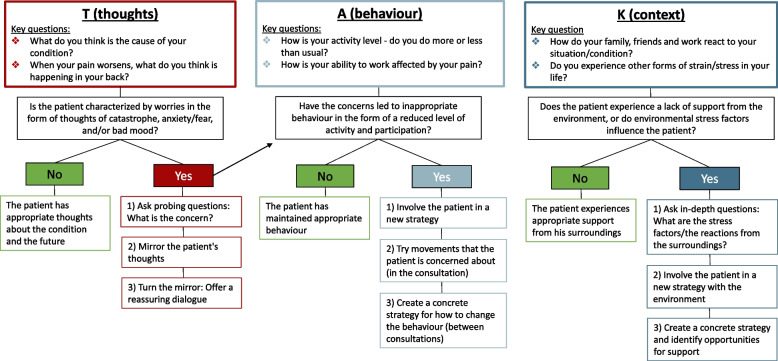
Specification of the target behaviour using the developed TAK model

The target behaviours *screening of psychosocial risk factors and offering patient education* were specified into a model named “Thanks for asking”, with the Danish word for thanks being 'TAK'. At the same time, TAK is an acronym for the Danish words for thoughts (Tanker), behaviour (Adfærd) and context (Kontekst). For an overview of the model, see Fig. 3.

Using the TAK model, the target behaviour was specified. As shown in Fig. 3, the TAK model guides the HCPs through screening and patient education within all three domains (i.e. thoughts, behaviour and context), elaborating on specific questions and suggesting strategies for involving the patients. See the model for details.

#### Identification of what needs to change: what barriers and facilitators to address

**Fig. 4:**
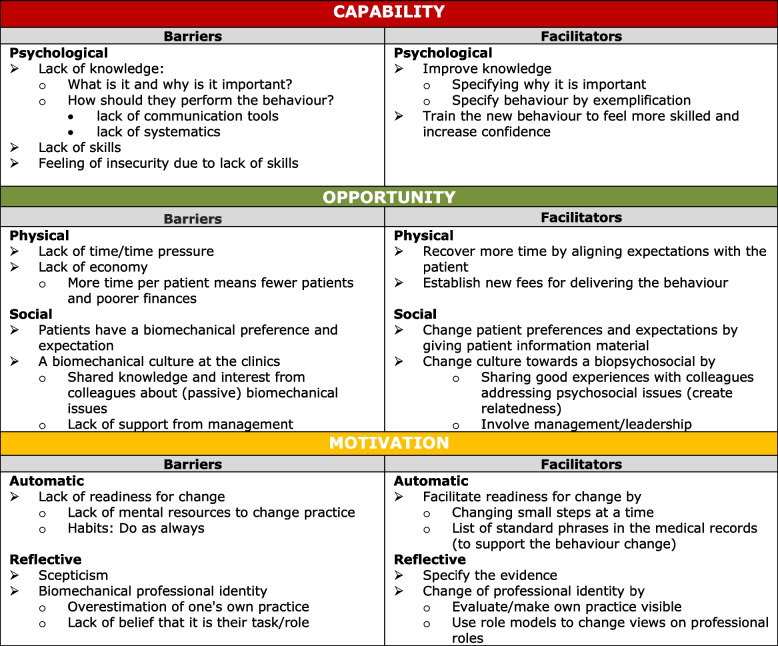
The participants’ identified barriers and facilitators for implementing the target behaviour

The participants identified what needed to change to implement the two target behaviours using the COM-B components as a guide (see Fig. 4). The participants expressed that the primary challenges in performing the target behaviour were due to a lack of knowledge and skills (i.e. capability), which is required to feel more confident in providing the target behaviour. Also, the participants highlighted that the HCPs’ opportunity to conduct the behaviour was affected both by time pressure and by a perception that patients prefer and expect a biomechanical approach. To promote the performance of the target behaviour in clinical practice, the participants suggested that HCPs should discuss this with their patients to align expectations. A biomechanical culture at the clinics was also emphasised as a challenge as it could negatively affect the feeling of relatedness with colleagues in terms of having a biopsychosocial approach. Engaging the whole clinic and promoting leadership was considered critical for successful implementation. The motivation for changing behaviour was further influenced by a lack of readiness to change and a biomechanical identity. These challenges were suggested addressed by implementing the change in small steps and having the HCPs reflect on their core roles.

### Stage 4: Design of the implementation programme

#### Identified intervention functions and behaviour change techniques

The identified barriers and facilitators listed for each COM-B component were linked with six BCW intervention functions (education, training, enablement, environment restructuring, modelling, persuasion) and the 24 behaviour change techniques (see Table 4). In summary, the participants suggested a flexible course of education and training to address the need for more knowledge, skills, and confidence. Likewise, persuasion and modelling were pointed out to address the need for more knowledge on why to perform the behaviour (reducing scepticism towards the validity of the guidelines) [28]. They advised using concrete communication exercises presented by short e-learning videos and supporting material explaining how to perform the target behaviour. Addressing the time pressure, the participants recommended the intervention function: enablement by offering the HCP education and training to align expectations with their patients. Patients’ preferences and the biomechanical culture were suggested to be changed by enablement and environmental restructuring by increasing the sense of relatedness with colleagues regarding having a biopsychosocial approach at the clinics. To promote a readiness to change, the participants requested a step-by-step implementation conducted in the clinics. Implementing the new behaviour in small steps would enable the HCPs to change habits as the new behaviour would be presented as “easy-to-perform tasks”, thus making it more applicable. Likewise, selecting a champion at every clinic as a support person was mentioned as an option for enabling a behaviour change. Interventions to increase the HCPs’ reflection on their role and behaviour were stated as a critical way to address the biomechanical professional identity. Several intervention functions were recommended to facilitate reflection: modelling, using a role model to demonstrate the new behaviour, education, persuasion and training to get feedback on one’s own behaviour and environmental restructuring to improve relatedness and facilitate acceptance of the new behaviour.

**Table 4:** The design process from the selected target behaviour to the final implementation programme

Target behaviour: Screening of psychosocial risk factors and offering patient education					
Barriers	Strategies	Mechanism/content	BCT	Functions	COM-B
Lack of education and knowledge	Webinar	Increase knowledge through education by providing information about the evidence, why the behaviour is essential, and how to perform the behaviour	Information about health + emotional consequences	Education/ Persuasion	Psychological Capability
			Credible source	Persuasion	
	E-learning video		Instruction on how to perform the behaviour	Education	
			Demonstration of the behaviour	Modelling	
Lack of skills and tools	E-learning videos	Improving skills by: Providing a demonstration of how to perform the behaviour	Demonstration of behaviour	Modelling	
			Instruction on how to perform the behaviour	Training	
	Communication exercise	Training the communication skills several times in small steps in a safe environment	Behavioural practice/rehearsal		
			Social support	Enablement	
	Peer learning	Giving and receiving feedback on behaviour to facilitate further development of skills	Feedback on behaviour	Education/ Persuasion/ Training	
Feeling unconfident	E-learning videos	Increase confidence by: Providing a demonstration of the new behaviour and telling that they can successfully perform the behaviour	Instruction on how to perform the behaviour	Education	
			Demonstration of the behaviour	Modelling	
			Verbal persuasion about capability	Persuasion	
	Communication exercise	Training the communication skills in small steps in a safe environment where the performance is not yet necessary	Behavioural practice/rehearsal	Training	
			Social support/ Exposure	Enablement/ Environmental restructuring	
Lack of time *and finances*	E-learning videos	Educate the behaviour to sharpen the communication	Instruction on how to perform the behaviour	Education/ Enablement	Physical Opportunity
	Communication exercise	Training expectation to align the time spent on communication	Behavioural practice/rehearsal	Training	
			Social support	Enablement	
	Group dialogue meetings	De-implement old habits to give more time for new behaviour + optimise patients' records	Habit reversal / substitution	Training	
			Restructuring the social environment	Enablement/ Environmental restructuring	
Biomechanical culture	Webinar	Changing culture by: Initiate a common understanding/knowledge and belief about performing the behaviour	Social support	Enablement	Social opportunity
			Framing/ reframing	Persuasion	
	Communication Exercises	Training skills together with colleagues in order to facilitate relatedness in performing the new behaviour	Restructuring the physical environment	Enablement/ Environmental restructuring	
	Group dialogue meetings	Changing environment by creating a common terminology and experience being able to share knowledge about not only biomechanical issues but also psychosocial factors	Restructuring the social environment		
			Social support		
Biomechanical patient expectation	Webinar + E-learning videos	Education of how to address patient expectation	Instruction on how to perform the behaviour	Education	Social opportunity
			Demonstration of the behaviour	Modelling	
			Information about others’ approval	Persuasion	
	Communication exercise	Training of how to address patient expectation	Behavioural practice/ rehearsal	Training	
	Peer learning	Providing information on behaviour facilitating a reflection of own behaviour and role	Feedback on behaviour	Education/ Persuasion/ Training	
	Group dialogue meetings	Enhance relatedness by sharing successes of new behaviour	Social support	Enablement	
			Focus on past success	Persuasion	
Lack of readiness to change	Implementation support	Enable behaviour change by supporting new behaviour with planned activities, pre-printed materials, reminders and a person to ask questions	Action planning	Enablement	Automatic Motivation
			Social support		
			Restructuring the physical environment	Enablement/ Environmental Restructuring	
	Stepwise implementation	Setting easy-to-perform tasks and adding more tasks (new behaviour) slowly to make it applicable to practice	Graded tasks	Enablement/ Training	
Habits	Stepwise implementation	Change old habits by introducing new behaviour in small affordable steps	Graded tasks		
	Communication exercises	Change habits by prompt rehearsal and repetition of the new behaviour	Habit formation	Training	
Biomechanical professional identity	Webinar + E-learning videos	Change professional identity by: Providing information about why and demonstrating how to perform the behaviour to initiate reflection on own role	Demonstration of the behaviour	Modelling	Reflective Motivation
			Instruction of how to perform the behaviour	Education	
	Communication exercises	Improving skills by practising the behaviour	Behavioural practice/ rehearsal	Training	
	Peer learning	Giving and receiving feedback on behaviour initiating a reflection of own behaviour and role	Feedback on behaviour	Education/ Persuasion/ Training	
			Discrepancy between current behaviour and goal		
			Social reward		
	Group dialogue meetings	Improve relatedness and facilitate acceptance of new behaviour. Also to provide practical help in carrying out the new behaviour by working together on journal standards	Social support (practical and emotional)	Enablement/ Environmental Restructuring	
			Restructuring the social environment		
Scepticism regarding evidence validity	Webinar	Increase belief about the importance by providing information about the evidence why the behaviour is essential, thereby increasing the motivation	Information about health + emotional consequences	Education/ Persuasion/ Modelling	Reflective Motivation
	E-learning video		Credible source	Persuasion	

*BCT* behaviour change techniques, *COM-B* Capability, Opportunity, Motivation – Behaviour

#### Identified mode of delivery

Concerning who and how the programme was to be delivered, an agreement was made to invite a keynote speaker to present the content of the target behaviour, while the first author, MHH, should deliver the various exercises/activities and to take on the role of the implementation consultant. The majority favoured a proposal to keep the intervention in the clinics and make the material as flexible as possible by making e-learning videos. Several participants recommended intervening in a joint effort at the clinics, including trying to engage the managers at the clinics.

**Fig. 5:**
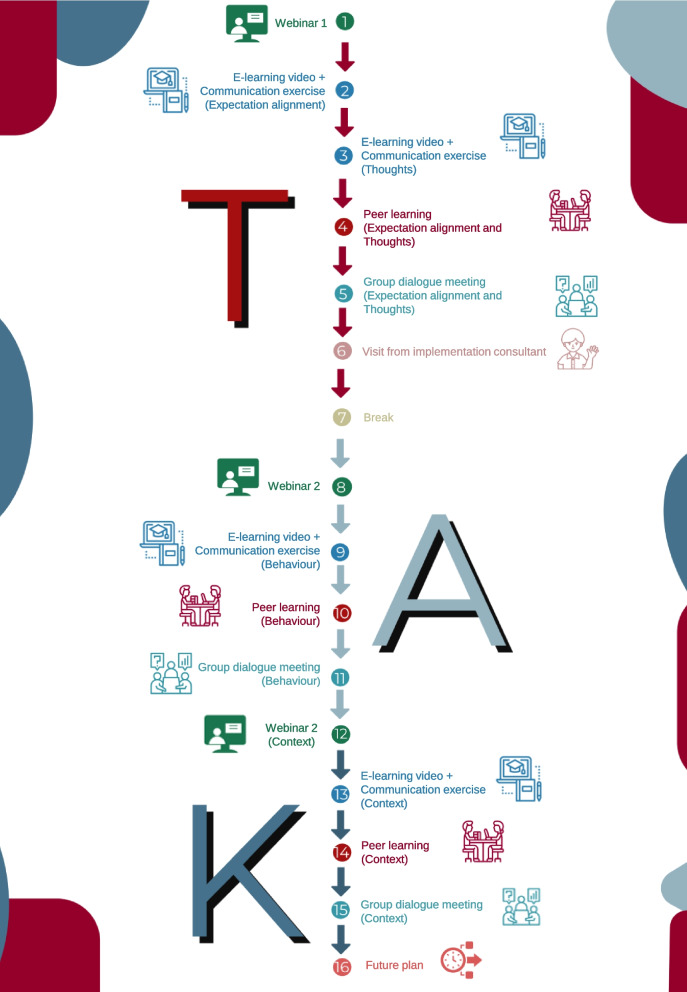
A roadmap showing the mode of delivery for the implementation programme

Regarding how often the programme should be delivered, the participants recommended implementing the new target behaviour in small steps. They had experienced too many overloaded weekend courses and acknowledged that a different approach was needed to be more effective for a behaviour change to occur. The use of 1 h per week would be manageable for most, and a presentation about “only” needing to use the equivalent of a 2-day course (16 h) spread over 4 months was concluded as an applicable delivery (See Fig. 5).

### Stage 5: The final implementation programme

The final implementation programme consisted of five strategies: [1] Webinars, [2] E-learning videos, [3] Communication exercises, [4] Peer learning, [5] Group dialogue meetings. In addition to the five strategies, the final implementation programme consisted of implementation support: champions, a physical material folder containing weekly descriptions of the activities, a weekly email reminder of the coming week’s activities, a specially designed website holding all intervention material [54], including the e-learning videos and a visit from an implementation consultant. Likewise, a “future plan” meeting was developed to support maintaining the behaviour. The content of the meeting consisted of deciding which strategies should be maintained after the intervention, how they should be held, and who was responsible. Following the GUIDED guideline, the description of the implementation programme is reported using a template by Proctor et al. [55], as shown in Table 5.

**Table 5:** Specification of the developed implementation strategies

Name	Definition	Actor	Action	Action Target	Temporality	Dose	Outcomes	Justification
Webinar	Education meeting and modelling	The responsible researcher of the project (MHH) and three different keynote speakers	Increase knowledge regarding new behaviour, the evidence behind the behaviour, why it is essential (consequences for patients) and how to perform the behaviour	Address lack of knowledge and scepticism by gaining knowledge about why the behaviour is essential, thereby also facilitating a more bio-psychosocial professional identity	Two webinars were conducted: Webinar 1 at week 1 and Webinar 2 at week 12. Both webinars were recorded and could be accessed on the homepage during the 16-week intervention	Webinar 1 lasted 1.5 h, and webinar 2 lasted 1 h	Acceptability applicability and feasibility	Theoretical evidence from the Behaviour Change Wheel (BCW) and participant involvement
E-learning videos	Education videos and persuasion	All participants, individually or in small groups at the clinics	Demonstration of behaviour by watching the videos and subsequently performing the communication exercises	Gain knowledge about how the new behaviour is carried out and change professional identity by reflecting on one's role	During the intervention at weeks 2, 3, 9 and 13	Each video lasted 5–15 min.	Acceptability and feasibility	Theoretical evidence from BCW and participant involvement
Communication exercises	Training of new behaviour	All participants conducting the exercises in small groups at the clinics	Training the communication either as role-play or as a dialogue exercise	Train and enable the new behaviour to feel more skilled and secure in practising it. By performing the exercises in groups, an environmental restructuring is promoted.	During the intervention at weeks 2, 3, 9 and 13	Each exercise took 30–45 min	Applicability and feasibility	Theoretical evidence from BCW and participant involvement
Peer learning	Peer feedback on new behaviour	All participants conducting peer learning in small groups	Observing patient consultation and giving/receiving feedback on the new behaviour	Giving/receiving feedback on new behaviour to address a lack of skills, biomechanical professional identity and culture	During the intervention at weeks 4, 10 and 14	Each peer learning session lasted 1 h	Acceptability applicability and feasibility	Theoretical evidence from BCW and participant involvement
Group dialogue meetings	Knowledge-sharing and medical records improvements	All participants at every clinic	Share knowledge and information about challenges and successes (approvals) and patient records	Restructuring the environment by knowledge sharing and modelling communication around psychosocial factors	During the intervention at weeks 5, 11 and 15	Each meeting lasted 1 h	Acceptability applicability and feasibility	Theoretical evidence from BCW and participant involvement
Implementation support: Champions	Facilitating behaviour change	Champion: One physiotherapist or chiropractor from every clinic	Ensuring practical issues such as booking appointments in the calendars and influencing habits by setting up reminder posters.	To enable and persuade a behaviour change by giving material and social support and adding new reminder objects to the environment	Before and during the intervention	The champions participated in a 2-h introduction meeting	Applicability and feasibility	Empirical and theoretical evidence from other studies shows the importance of having a champion associated

## Discussion

This paper describes the important development phase of a research project developing and evaluating a tailored implementation programme targeting guideline implementation within LBP care. A thorough selection and description of the target behaviours were carried out. The process revealed that the two professions (physiotherapists and chiropractors) strongly agreed that screening the patient’s psychosocial risk factors and offering patient education were the most critical guideline recommendations for the programme to target. The barriers and facilitators for the selected behaviours were successfully described using COM-B, and a multi-pronged implementation programme was designed to address the identified barriers and facilitators.

### Discussion of the five stages in the development process

Several studies have evaluated the effect of implementation strategies for implementing LBP guidelines among HCPs on patients [6, 33, 56, 57] and HCPs [32, 33, 36, 56–58]. However, to our knowledge, only four studies have described the development process of their implementation programmes [59–62], suggesting that these development studies are needed in the field of implementation programme evaluation. The challenges imposed by a non-transparent development process are elaborated below.

#### Establish a theoretical frame to guide the development process

A challenge when assessing the effectiveness of multi-component strategies is knowing which strategies led to increased implementation effectiveness (how and why the strategies work). In the present study and the four previous development studies [59–62], the hypothesis about mechanisms of change is based on behaviour change theory, which might contribute to the knowledge of how or why the strategies (did not) work [45]. However, a review of the use of theory in the design of guideline dissemination and implementation strategies showed poor use of theory [63]. The poor theoretical underpinning makes understanding and explaining “the black box” challenging. It leaves implementation science without an opportunity to explore potential causal mechanisms that predict the likelihood of implementation success and develop improved strategies to achieve more successful implementation [64]. In our study, using BCW (including COM-B) led to the identification of a broad spectrum of barriers and facilitators for implementing the target behaviour. The theory further enabled an understanding of the mechanisms of how the identified barriers and facilitators were best influenced and thereby helped to determine proper intervention functions.

#### Involve participants

Through a co-design process involving participants (i.e. HCP implementers) already in the development phase, the programme could be tailored to the contextual barriers and facilitators. Even though co-design appears widely used, it is seldom described. As a consequence, the knowledge of *when* and *how* to use involvement remains unclear [50]. In the present study, the co-design process was planned to be carried out as four workshops in the context where the target behaviour was to be implemented (i.e. primary care clinics), as several researchers have emphasized this as significant [50]. Although it was only possible to host two workshops at the participants’ clinics due to the Covid-19 pandemic, this may still have greatly impacted building a trustful relationship with the participants [65].

Another crucial factor in a co-design process is facilitating participant engagement to ensure a well-functioning collaboration between the research team and stakeholders [65]. Workshops are a less frequently used activity in co-design [50], but is a format that has been argued to enhance engagement among stakeholders [65], which is something that was also observed in our study. Indeed, the interaction between the individual completion of the worksheets, the group work and the joint discussions prompted dynamic communication and a positive and trusting environment with a high degree of engagement. Beyond that, the format also challenged the research group to ensure the equal influence of all participants, as some participants were quieter than others. Here, the research team strived to facilitate equality by grouping more introverted participants with participants with a high degree of inclusive and listening skills. Likewise, the research team tried to ensure inclusion by asking participants who had yet to share their thoughts for input in the joint discussions.

The power balance was also challenged by some participants who automatically took the lead in the group discussions because of their expert knowledge of the target behaviour. Research has shown that one technique to initiate power balance is articulating the participants’ power before starting the co-design process [65, 66]. This technique was used in the present study, where the participants at the first workshop were asked to share their thoughts on the role and expectations for their participation. The participants’ giving words to their views on power allowed the researchers to address the sense of insecurity by some participants about whether they had the requested knowledge, skills and experience to be able to contribute to the development process. The research team emphasized the value of the expertise of each participant representing the target population.

#### Understand the behaviour

The participants’ selection of the target behaviours was in accordance with the findings of a study by Driver et al., showing that Australian physiotherapists reported it was important to have knowledge about psychosocial interventions and considered it equally important to be able to offer such interventions [67].

Also, the barriers and facilitators to implementing a biopsychosocial approach found in the present study were in line with other studies [29, 68]. However, whether all necessary barriers have been identified has yet to be determined. A study by Mescouto et al. showed that institutional contexts reinforce the power of biomedical norms for physical therapy practice, which makes them difficult to change [69]. This includes not only the individual physiotherapy clinic but also the economic and healthcare system where physical therapists and patients are nested [69]. Thus, identifying and addressing institutional barriers and facilitators might be important to achieve implementation success. However, including the institutional level to a higher degree was beyond the scope of this study.

#### Design the implementation programme

Although several typologies of implementation strategies have been proposed [70], a systematic approach to developing and selecting these strategies has yet to be recommended. No pattern exists in what works for whom [34], and many implementation strategies have been used across studies; over 70 discrete implementation strategies (e.g. education, audit and feedback, reminders, peer learning) have been identified [70, 71], in addition to the diversity of environments in which they have been used. The implementation programme in this study was designed as a multi-pronged programme consisting of five strategies. Several studies have found no correlation between the number of strategies and the effect of strategies [72, 73]; however, a systematic review examining which strategies are most effective in changing clinical practice for non-specific LBP has shown single interventions to be largely unsuccessful [36]. The result of the review supports the development of the multi-pronged programme in the present study.

The extent of the multi-pronged programme was designed based on the participants’ experiences and a pragmatic decision regarding how many resources were available. Research shows that the extent of educational and training interventions required to deliver biopsychosocial elements to patients with LBP differs between studies [74, 75]. A review by van Erp et al. concluded that a short 2-day exercise programme by physical therapists in one study improved patient outcomes but that a 4-day exercise programme in another study did not [74, 76, 77]. A review by Mesner et al. concluded that increased frequency and duration of implementation interventions lead to greater success [36]. Likewise, studies show that interventions with time allocated between learning sessions will likely improve clinical skills, allowing HCPs to adopt the new behaviour in the relevant context [78]. Increased frequency and time between the strategies support the present study’s design.

#### Final implementation programme

The programme in the present study necessitated a paradigmatic shift in beliefs and behaviour among the HCPs, moving away from a primarily biomechanical professional identity and culture towards a more biopsychosocial approach that aligns better with the evidence in the field. The difficulty of incorporating the biopsychosocial paradigm suggests its complexity and that it is embedded not only in the individual HCP but also in the culture and daily practice of the given healthcare system [79]. Empirical findings suggest repeated behaviours in constant contexts are challenging to change [80]. However, in trying to do so, a change in habits can be facilitated by a change in culture. Therefore, interventions that focus on changing the context to break the maintenance of existing habits are more likely to succeed [80]. Here, the step-by-step design of our implementation programme is supported in the literature [80]. The success of the developed implementation programme in terms of creating a change in the targeted behaviour in practice is presently being evaluated and will be reported in a separate paper. The design of this study (theory-driven and explicit functions) may help elucidate which strategies in the programme are effective.

### Methodological discussion

A strength of this study was the use of theory to guide the behaviour change process, as it provided perceptive knowledge on essential components and thereby optimised the chance for the implementation programme to be effective. Another strength was the involvement of participants. Developing an implementation programme is challenged by fully understanding the target behaviour and the context in which the behaviour needs to change. Thus, ensuring the integration of theory and evidence with the experiences and perspectives of participants gave an exhaustive insight into the understanding of the behaviour. Gathering two professional groups gave the study the necessary knowledge about whether two different strategies should be designed. Thus, another strength of this study was that the programme could be applied to both professions. Also, achieving diversity in demographic variables in the sampling was a strength as it ensured multiple perspectives were represented. A limitation of the study was that only one of the co-authors (CBR) had experience with running user-involvement and co-design processes prior to this study. However, extensive preparation, by applying theory to guide the process and by recording and observing, e.g. group dynamics, allowed the research team to reflect on the challenges occurring and adjust the content and format of the subsequent workshop.

## Conclusion

The development process of a contextually tailored programme for implementing the LBP guideline recommendations was described. The development was based on behaviour change theory and was co-designed through four workshops involving physiotherapists and chiropractors from primary care clinics. The final implementation programme comprised five strategies aiming to overcome the identified barriers and facilitators. Whether the final programme will enhance evidence-based LBP care is presently being evaluated and will be published in a separate paper.

### Supplementary Information

**Supplementary material 1.**

**Supplementary material 2.**

**Supplementary material 3.**

**Supplementary material 4.**

**Supplementary material 5.**

## Data Availability

Not applicable. Due to the European GDPR, making data available is impossible, as it would require distortion of images and sound. Also, all audio files, videos, posters, etc., are in Danish, and it is therefore impossible to make them comprehensibly accessible to the readers of Implementation Science.

## Abbreviations

BCW: Behaviour Change Wheel
COM-B: Capability, Opportunity, Motivation - Behaviour
HCPs: Healthcare professionals
LBP: Low back pain
