# Supplementary material for: Developing a low back pain guideline implementation programme in collaboration with physiotherapists and chiropractors using the Behaviour Change Wheel: a theory-driven design study
Source: Implement Sci Commun. 2024 Apr 3;5:33. doi: 10.1186/s43058-024-00568-x (PMC10993475; doi:10.1186/s43058-024-00568-x)
Supplement: Supplementary file 2 — Supplementary material 2. [file 43058_2024_568_MOESM2_ESM.pdf]

**Recommendations in the guidelines**	Are the consequences of not applying the recommendation serious?	Are there many of your colleagues who do not use the recommendation?	Is the recommendation possible to apply in practice?	Is it important to implement the recommendation in practice?	**Total score**
**Assessment**					
Screening of psychosocial risk factors					
Do not offer routine imaging					
**Treatment**					
Supervised training in addition to usual treatment					
Manual joint mobilisation in addition to usual treatment					
Do not offer routine acupuncture					
Do not offer routine pain medication (paracetamol, NSAIDs, opioids)					
**Information and guidance**					
Advise to stay physically active					
Advise to stay or return to work as soon as possible					
Offer patient education and reassuring information about the benign nature and prognosis of LBP					

Scoring: 1: no, 2: probably not, 3: do not know, 4: probably yes, 5: yes.
