# Supplementary material for: Developing a low back pain guideline implementation programme in collaboration with physiotherapists and chiropractors using the Behaviour Change Wheel: a theory-driven design study
Source: Implement Sci Commun. 2024 Apr 3;5:33. doi: 10.1186/s43058-024-00568-x (PMC10993475; doi:10.1186/s43058-024-00568-x)
Supplement: Supplementary file 3 — Supplementary material 3. [file 43058_2024_568_MOESM3_ESM.pdf]

**Recommendations/ Target behaviour**	What should the behaviour include?	How should the behaviour be practised?	When and how often should the behaviour be performed?	To whom should the behaviour be performed?
**Screening of psychosocial risk factors**				
**Patient education and reassuring information**				
