# Supplementary material for: Developing a low back pain guideline implementation programme in collaboration with physiotherapists and chiropractors using the Behaviour Change Wheel: a theory-driven design study
Source: Implement Sci Commun. 2024 Apr 3;5:33. doi: 10.1186/s43058-024-00568-x (PMC10993475; doi:10.1186/s43058-024-00568-x)
Supplement: Supplementary file 4 — Supplementary material 4. [file 43058_2024_568_MOESM4_ESM.pdf]

NAME________________________

Barriers and facilitators of the behaviour: Screening of psychosocial factors

**What changes are necessary to achieve desired behaviour: Screening for psychosocial factors?**<br>-is de-implementation necessary?		
**COM-B components**	**Barriers to the behaviour**<br>-what challenges are there with the new behaviour?	**Facilitators of the behaviour**<br>-how is the new behaviour supported?
**Capacity**<br>Do the clinicians have the necessary "physical ability" and the knowledge and skills to screen for psychosocial risk factors?		
**Motivation**<br>Are the clinicians motivated to screen the patients' psychosocial risk factors? Do they have the awareness/belief that screening for psychosocial risk factors is important in treating patients with back pain?		
**Opportunities**<br>Do the clinics have the physical framework (time, confined spaces) to screen for psychosocial risk factors? Do the clinics have support and understanding from the environment, e.g. managers and colleagues, to screen for psychosocial risk factors?		
