# Supplementary material for: Developing a low back pain guideline implementation programme in collaboration with physiotherapists and chiropractors using the Behaviour Change Wheel: a theory-driven design study
Source: Implement Sci Commun. 2024 Apr 3;5:33. doi: 10.1186/s43058-024-00568-x (PMC10993475; doi:10.1186/s43058-024-00568-x)
Supplement: Supplementary file 5 — Supplementary material 5. [file 43058_2024_568_MOESM5_ESM.docx]

| **Name it** | **Define it** | **Actor** | **Action** | **Action Target** | **Temporality** | **Dose** | **Outcomes** | **Justification** |
| --- | --- | --- | --- | --- | --- | --- | --- | --- |
| Webinar | Education meeting and modelling | The responsible researcher of the project (MHH) and three different keynote speakers | Increase knowledge regarding new behaviour, the evidence behind the behaviour, why it is essential (consequences for patients) and how to perform the behaviour | Address lack of knowledge and scepticism by gaining knowledge about why the behaviour is essential, thereby also facilitating a more bio-psychosocial professional identity | Two webinars were conducted: Webinar 1 at week 1 and Webinar 2 at week 11.  Both webinars were recorded and could be accessed on the homepage during the 16-week intervention | Webinar 1 lasted 1.5 hours, and webinar 2 lasted 1 hour | Acceptability applicability and feasibility | Theoretical evidence from the Behaviour Change Wheel (BCW) recommends education as a helpful intervention function for addressing the lack of knowledge |
| E-learning videos | Education videos and persuasion | All participants, individually or in small groups at the clinics | Demonstration of behaviour by watching the videos and subsequently performing the communication exercises | Gain knowledge about how the new behaviour is carried out and change professional identity by reflecting on one's role | During the intervention at week 2, 3, 9 and 12 | Each video lasted 5-15 minutes. | Acceptability and feasibility | Theoretical evidence from BCW recommends education as a proper intervention function for addressing the lack of knowledge |
| Communication exercises | Training of new behaviour | All participants conducting the exercises in small groups at the clinics | Training the communication either as role-play or as a dialogue exercise | Train and enable the new behaviour to feel more skilled and secure in practising it. By performing the exercises in groups, an environmental restructuring is promoted. | During the intervention at week 2, 3, 9 and 12 | Each exercise took 30-45 minutes | Applicability and feasibility | Theoretical evidence from BCW recommends training as a useful intervention function for addressing a lack of skills |
| Peer learning | Peer feedback on new behaviour | All participants conducting peer learning in small groups | Observing patient consultation and giving/receiving feedback on the new behaviour | Giving/receiving feedback on new behaviour to address a lack of skills, biomechanical professional identity and culture | During the intervention at week 4, 10, and 13 | Each peer learning session lasted 1 hour | Acceptability applicability and feasibility | Theoretical evidence from BCW recommends enablement as a helpful intervention function for addressing motivation and opportunities |
| Group dialogue meetings | Knowledge-sharing and records improvements | All participants at every clinic | Share knowledge and information about challenges and successes (approvals) and patient records | Restructuring the environment by knowledge sharing and modelling communication around psychosocial factors | During the intervention at week 5, 11 and 14 | Each meeting lasted 1 hour | Acceptability applicability and feasibility | Pragmatic evidence from participants |
| Implementation support: Champions | Facilitating behaviour change | Champion: One physiotherapist or chiropractor from every clinic | Ensuring practical issues such as booking appointments in the calendars and influencing habits by setting up reminder posters. | To enable and persuade a behaviour change by giving material and social support and adding new reminder objects to the environment | Before and during the intervention | The champions participated in a 2-hour introduction meeting | Applicability and feasibility | Empirical and theoretical evidence from other studies shows the importance of having a champion associated |
